# The Bolingbroke Hospital, Wandsworth Common

**Published:** 1901-06-15

**Authors:** 


### THE BOLINGBROKE HOSPITAL, WANDSWORTH COMMON.

### THE EXTENSION SCHEME.

A remarkable evidence of the popularity of the Bolingbroke Hospital at Wandsworth Common among the working classes was afforded on Saturday on the occasion of the ceremony of laying the foundation stone of the new out-patient department, as a memorial to Queen Victoria. The time fixed for the function was half-past three in the afternoon, but it was nearly four o'clock before the Lord Mayor of London, who had undertaken to be the principal performer, arrived at the scene of action. The delay was not the result of want of punctuality on the part of the Lord Mayor; it was due entirely to a block in the streets caused by a long procession of trade and friendly societies who went to meet the civic party about a mile away from the hospital, and accompanied it to the site.

Meanwhile, a large number of invited guests had assembled in the pleasant grounds attached to the hospital. The weather being brilliantly fine, some of the patients were able to witness the ceremony, and the nurses were very much in evidence. The arrival of the Lord Mayor, who was accompanied by the Lady Mayoress, Alderman and Sheriff Morgan, Alderman and Sheriff Lawrence, M.P., the City Marshal, and other officials, was the signal for a round of cheering.

The President of the Board of Governors, Canon Erskine Clarke, Vicar of Battersea—who, at his own risk, but with the aid of contributions, purchased Bolingbroke House, nearly twenty years ago, and opened it as a self-supporting hospital—briefly addressed the Lord Mayor in explanation of the position. Battersea, he said, contained seventy miles of streets, and the Bolingbroke Hospital, with only thirty beds, was totally inadequate to meet the demands of the district it was called upon to serve. It was the only provision for the treatment of accidents in the vast area comprising Battersea, Wandsworth, Balham, Tooting, and Earlsfield, with a population of nearly half a million people, the nearest general hospital being four miles distant. The number of accidents treated last year was upwards of 3,000 involving more than 15,000 attendances; and 255 persons were retained, in consequence of the serious nature of their cases, for periods varying from a few days to many months. He pointed out that, not only the rapid increase of the population in the districts named, but the enormous traffic at the local railway stations, and the continued addition of new factories to those already at work, had augmented the number of accidents to be treated, and proved the necessity of a vigorous effort to grapple more adequately with the requirements of the locality. In the course of his speech Canon Clarke paid a warm tribute to the work of the medical superintendent, Mr. Cecil Lyster, the matron Miss Edith Thompson, and the staff.

The Bishop of Southwark, who was vested in his episcopal robes, having offered prayers, the Lord Mayor proceeded to make use of the trowel in a workmanlike fashion, and as soon as he had declared the foundation to be "well and truly laid," the presentation of purses in aid of the building fund to the Lady Mayoress (Miss Green) was commenced, the first donor being a baby in arms, who behaved exceedingly well.

Mr. Percy Thornton, M.P., next moved, and Mr. H. Kimber M.P., seconded, a vote of thanks to the Lady Mayoress and purse-holders. To the member for Clapham the company were indebted for an extremely interesting historical sketch of Bolingbroke Grove. The Mayors of Battersea and Wandsworth were appropriately charged with the duty of proposing and seconding a vote of thanks to the Lord Mayor, who, in acknowledging the vote, strongly commended the object of the gathering, that of honouring the memory of a great and good Queen, and of benefiting suffering humanity.

During the whole of the ceremony the heat of the sun was excessive, and it was not without relief that, as soon as the National Anthem had been sung with enthusiasm, the guests retired into the hospital to partake of tea. In the evening the grounds were illuminated, and an *al fresco* concert was given.

The cost of the out-patient department will be upwards of £5,000, towards which £3,246 had been subscribed before the laying of the foundation-stone. The Governors anticipate that the new building will be ready for occupation in the autumn, and it may be hoped that it will be opened free of debt.

